# Replication Kinetics of *Rickettsia raoultii* in Tick Cell Lines

**DOI:** 10.3390/microorganisms9071370

**Published:** 2021-06-24

**Authors:** Nurul Aini Husin, Jing Jing Khoo, Mulya Mustika Sari Zulkifli, Lesley Bell-Sakyi, Sazaly AbuBakar

**Affiliations:** 1 Tropical Infectious Diseases Research and Education Centre, Level 2, High Impact Research Building, Universiti Malaya, Kuala Lumpur 50603, Malaysia; nurulainihusin@yahoo.com (N.A.H.); khoojj@gmail.com (J.J.K.); mulyamustika@um.edu.my (M.M.S.Z.); 2 Institute for Advanced Studies, Universiti Malaya, Kuala Lumpur 50603, Malaysia; 3 Department of Infection Biology and Microbiomes, Institute of Infection, Veterinary and Ecological Sciences, University of Liverpool, Liverpool L3 5RF, UK; L.Bell-Sakyi@liverpool.ac.uk; 4 Department of Medical Microbiology, Faculty of Medicine, Universiti Malaya, Kuala Lumpur 50603, Malaysia

**Keywords:** vector-borne disease, *Rickettsiaraoultii*, infectious disease, tick cell line

## Abstract

*Rickettsia raoultii* is one of the causative agents of tick-borne lymphadenopathy in humans. This bacterium was previously isolated and propagated in tick cell lines; however, the growth characteristics have not been investigated. Here, we present the replication kinetics of *R. raoultii* in cell lines derived from different tick genera (BME/CTVM23, RSE/PILS35, and IDE8). Tick cell cultures were infected in duplicate with cryopreserved *R. raoultii* prepared from homologous cell lines. By 12–14 days post infection, 100% of the cells were infected, as visualized in Giemsa-stained cytocentrifuge smears. *R. raoultii* growth curves, determined by rickettsiae-specific gltA qPCR, exhibited lag, exponential, stationary and death phases. Exponential phases of 4–12 days and generation times of 0.9–2.6 days were observed. *R. raoultii* in BME/CTVM23 and RSE/PILS35 cultures showed, respectively, 39.5- and 37.1-fold increases compared to the inoculum. In contrast, multiplication of *R. raoultii* in the IDE8 cultures was 110.1-fold greater than the inoculum with a 7-day stationary phase. These findings suggest variation in the growth kinetics of *R. raoultii* in the different tick cell lines tested, amongst which IDE8 cells could tolerate the highest levels of *R. raoultii* replication. Further studies of *R. raoultii* are needed for a better understanding of its persistence within tick populations.

## 1. Introduction

*Rickettsia raoultii* is a spotted fever group rickettsial species, which was first described in *Dermacentor nuttalli* (reported then as genotypes DnS14 and DnS28) and *Rhipicephalus pumilio* (genotype RpA4) ticks from Siberia [1]. It was later described from *Dermacentor* spp. ticks, mainly *D. nuttalli*, *Dermacentor reticulatus*, *Dermacentor marginatus* and *Dermacentor silvarum*, from Russia [2], countries in Europe [2,3,4,5], and Asia [6,7,8,9]. *R. raoultii* has also been reported in *Haemaphysalis erinacei* [10] and *Ixodes ricinus* [11] ticks, and even in other arthropod species, such as *Melophagus ovinus* [12], suggesting a possible wider host range in addition to *Dermacentor* spp. ticks. *Rickettsia* spp., genetically related to *R. raoultii* (>98% identity in target genes), were also described from *Dermacentor* spp. ticks from Southeast Asian countries [13,14]. 

*R. raoultii*, together with *Rickettsia slovaca*, is a causative agent of tick-borne lymphadenopathy (TIBOLA), also known as *Dermacentor*-borne necrosis erythema and lymphadenopathy (DEBONEL) [15]. The syndrome is associated with a tick bite, an eschar at the tick bite site (frequently on the scalp) and cervical lymphadenopathies [15]. *R. raoultii* infections with erythematous rash and fever, but without lymphadenopathy, were also reported in patients from China [16]. Other reported clinical manifestations of *R. raoultii* infections include meningeal syndrome [17], and neurological abnormalities such as eyelid droop and high cerebrospinal pressure [18]. Although normally associated with mild infections, more severe infections with leukopenia, thrombocytopenia and septic parameters have also been reported, suggesting varying degrees of virulence for [19], or susceptibility to *R. raoultii*. Although TIBOLA/DEBONEL is attributed to both *R. slovaca* and *R. raoultii*, studies have suggested that *R. raoultii* is more highly prevalent among *Dermacentor* spp. ticks [15]. However, there have been more reports of *R. slovaca* infection, implying that *R. raoultii* is less pathogenic [15] or less frequently transmitted to humans. Despite its widespread presence in Europe, Russia, and Asia, information relating to the virulence and pathogenicity of *R. raoultii* is still scarce; more thorough characterization is necessary to understand these aspects of the bacterium.

*R. raoultii* was first isolated from *Dermacentor* spp. ticks into L929 and Vero cells by Mediannikov and co-workers [2]. Later, *R. raoultii* was also isolated into embryo-derived tick cell lines originated from *Rhipicephalus microplus* [20,21,22,23] and *Rhipicephalus sanguineus* [24]. The bacterium was also found to be able to infect cell lines derived from *Dermacentor albipictus* and *Dermacentor nitens* [20]. Since ticks are natural reservoirs and vectors of some *Rickettsia* spp., tick cell lines are a useful system for the isolation and propagation of *R. raoultii* from ticks or clinical samples for further investigation of its virulence and pathogenicity [20,24]. 

The purpose of this study was to establish the infection rates and replication kinetics of *R. raoultii* in three tick cell lines—BME/CTVM23 derived from *R. microplus* [20], RSE/PILS35 derived from *R. sanguineus* [25], and IDE8 derived from *Ixodes scapularis* [26]. The aim was to increase understanding of the growth characteristics of *R. raoultii* in tick cell lines and provide the basis for further studies into its invasiveness for host cells.

## 2. Materials and Methods

### 2.1. Maintenance of Tick Cell and R. raoultii Cultures

The tick cell lines and bacterial culture were obtained from the Tick Cell Biobank, University of Liverpool, UK and maintained in the Tick Cell Biobank Asia Outpost at TIDREC, Universiti Malaya, Malaysia. The *R. microplus*-derived cell line BME/CTVM23 at passage 77 [20] and *R. sanguineus*-derived cell line RSE/PILS35 at passage 16 [25] were grown at 32 °C and 28 °C, respectively, in L-15 (Leibovitz) medium supplemented with 10% tryptose phosphate broth (TPB), 20% fetal bovine serum (FBS), 2 mM L-glutamine and antibiotics (100 units/mL penicillin and 100 µg/mL streptomycin). The *I. scapularis-*derived cell line IDE8 at passage 98 [26] was maintained at 32 °C in L-15B medium [27] supplemented with 10% TPB, 5% FBS, 0.1% bovine lipoprotein (MP Biomedicals, Solon, OH, USA), 2 mM L-glutamine and antibiotics. All cell lines were maintained in sealed flat-sided culture tubes (Nunc, Thermo Fisher, Loughborough, UK) with ¾ of the medium replaced weekly and sub-culture performed at 1–3-month intervals.

*R. raoultii* (strain Białystok1) was provided in a culture maintained in the BME/CTVM23 cell line [21]. The bacterial culture was maintained following the conditions for BME/CTVM23 cells outlined above, but with incubation temperature at 28 °C instead of 32 °C. The *R. raoultii* was maintained by passaging the bacteria onto fresh BME/CTVM23 cells once every three weeks. 

### 2.2. Preparation of R. raoultii Stock Culture

Heavily infected BME/CTVM23 cells were resuspended by pipetting and forcibly passed through a 25 G needle 5 times to release the bacteria from the cells. The resulting suspension was filtered through a 2.0 µm membrane filter and centrifuged at 1000× *g* for 5 min at 4 °C, to remove intact cells and cell debris. Next, equal volumes of the suspension were used to inoculate IDE8 and RSE/PILS35 cell cultures. The inoculated cells were maintained at 28 °C and were observed daily for signs of cytopathic effects using a BMI-100 inverted microscope (Biobase, Shandong, China). Once severe cytopathic effects were observed (i.e., most cells rounded up or detached from the bottom of the culture tube, indicating heavy *R. raoultii* infection), aliquots of the infected cells were immediately cryopreserved in their respective culture medium with the addition of 10% dimethyl sulfoxide as described previously [21], to be used as the bacterial stock for infection studies. An aliquot of each of the infected cells was used for DNA extraction and PCR amplification of the tick-specific partial 16S rRNA sequence utilizing the primer pair 16S+1 and 16S−1 from a previously published protocol [28]. To exclude the possibility that any carry-over of live BME/CTVM23 cells into the recipient IDE8 and RSE/PILS35 cultures had occurred, the PCR amplicons were sequenced and subjected to NCBI BLAST analysis at 7 and 13 days post inoculation (dpi). 

### 2.3. Infection of Cells

One day prior to infection, duplicate cultures were set up in 2.2 mL culture medium with the following cell densities for each cell line to be tested: the BME/CTVM23 cells were seeded at 6 × 10^6^ cells/mL, RSE/PILS35 cells were seeded at 2 × 10^6^ cells/mL and IDE8 cells were seeded at 5 × 10^6^ cells/mL. To each culture, 200 µL of thawed, cryopreserved bacterial stock prepared from the homologous cell line was added. Post infection, the cell cultures were monitored daily for signs of cytopathic effects. Cells were collected at the indicated intervals for preparation of Giemsa-stained cytocentrifuge smears and for bacterial quantification by quantitative PCR (qPCR) as described below. Fresh medium was used to replace the volume that was removed when the cell suspension was taken from the culture tube.

### 2.4. Visualization of Bacteria by Giemsa Staining 

Cytocentrifuge smears were prepared from 50 µL of cell suspension centrifuged for 5 min at 1000 rpm. in a Cytospin 3 cytocentrifuge (Shandon, Pittsburgh, PA, USA). The resultant smears were air-dried, fixed in methanol for 3 min, stained with Giemsa (Merck, Darmstadt, Germany) and rinsed 3 times with water buffered to pH 7. The Giemsa-stained smears were examined under a compound microscope (GX Microscopes, Suffolk, UK) at 1000× magnification for the presence of bacteria. The GXCAM digital camera and GXCapture software were used to capture images of the cells. The percentage of infected cells was determined by calculating the number of infected cells × 100, divided by the total number of cells (at least 200 cells examined for each sample).

### 2.5. Quantification of Bacteria by qPCR

DNA was extracted from 200 µL of cell suspension collected from each time point of the experiment using a NucleoSpin^®^ tissue kit (Macherey-Nagel, Düren, Germany) following the manufacturer’s protocol. DNA was also extracted from a 200 µL aliquot of cryopreserved bacterial stock prepared from the homologous cell line as the representation of the number of bacterial DNA copies present at time point 0 dpi in the infection time course. A previously published qPCR protocol targeting a 74 base-pair fragment of the *Rickettsia* citrate synthase (*gltA*) gene [29] was used to quantify absolute numbers of *R. raoultii* in the infected cultures. The qPCR was performed using a CFX96 touch real-time PCR detection system (Bio-Rad, Watford, UK) with a 6-carboxyfluorescein (FAM) and black-hole quencher (BHQ1-) labelled TaqMan probe (Integrated DNA Technologies, Singapore). All reactions were prepared in 25 µL reaction volumes, comprising final concentrations of 1 × TaqMan fast advanced master mix (Applied Biosystems, Waltham, MA, USA), 200 nM of each primer and probe, and 1 µL DNA template. The PCR cycling conditions were as follows: initial holding temperature at 50 °C for 3 min, followed by 95 °C for 5 min and 40 cycles of 95 °C for 20 s and 60 °C for 40 s. To determine the bacterial DNA copy number, a pIDTSmart (Amp) vector (Integrated DNA Technologies, The Gemini Singapore science Park ll, Singapore) containing the gene target was used to construct a standard curve with serial dilution in the range of 1 × 10^5^ to 1 × 10^13^ copies. 

To determine the numbers of tick cells in the cultures, a qPCR assay targeting a 77 base-pair fragment of a tick single-copy nuclear gene, ribosomal protein L6 (*rpl6*) was performed as described previously [30]. Each reaction contained final concentrations of 1 × SensiFast SYBR no-ROX master mix (Bioline, UK), 200 nM each of forward and reverse primers and 1 µL DNA at a final volume of 20 µL. The reactions were conducted with an initial denaturation at 95 °C for 10 min, followed by 35 cycles of denaturation at 95 °C for 15 s, annealing at 55 °C for 30 s and extension at 72 °C for 15 s. Following amplification, a melt curve from 55 °C to 95 °C with increasing increments of 0.5 °C per cycle was examined to confirm that only a single target had been amplified. To determine the tick DNA copies, a synthesized gene target obtained from the Tick Cell Biobank was used to construct a standard curve with serial dilutions in the range of 5 × 10^−1^ to 5 × 10^6^ copies. The generation times for *R. raoultii* in each tick cell culture were calculated using the following equation [31]:

$$\mathrm{Mean}\;\mathrm{generation}\;\mathrm{time}\;=0.301t/\left(\log_{10}N_{t}-\log_{10}N_{0}\right)\;$$

where $N_{0}$ is the number of bacteria at the beginning of the exponential phase, $N_{t}$ is the number of bacteria at the end of the exponential phase and *t* is the interval between $N_{0}$ and $N_{t}$.

## 3. Results

### 3.1. Sequencing of the Tick-Specific Partial 16S rRNA Gene

Sequencing of the PCR amplicon from the inoculated IDE8 and RSE/PILS35 cells confirmed the absence of the *R. microplus* 16S rRNA sequence in either of the recipient cultures at 7 and 13 dpi. Therefore, this indicates that there was no carry-over of live BME/CTVM23 cells into the recipient cultures of the IDE8 and RSE/PILS35 cells.

### 3.2. Microscopic Observation of R. raoultii-Infected Tick Cells

**Figure 1:**
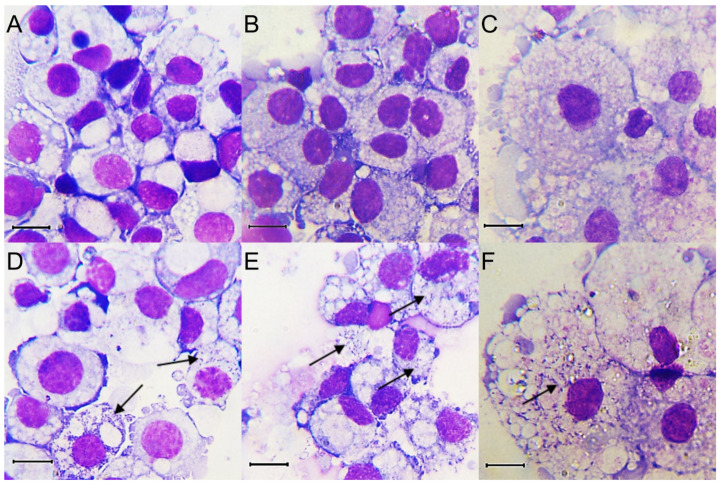
Giemsa-stained cytocentrifuge smears prepared pre-infection (**A**–**C**) and at 7 days post infection (**D**–**F**) with *Rickettsia raoultii* of tick cell lines BME/CTVM23 (**A**,**D**), IDE8 (**B**,**E**) and RSE/PILS35 (**C**,**F**). Arrows indicate the presence of bacteria. Scale bars represent 10 µm.

Duplicate cultures of each of BME/CTVM23, RSE/PILS35 and IDE8 cell lines were infected with *R. raoultii* and monitored at the selected time points by Giemsa-stained cytocentrifuge smears for the presence of bacteria. Noticeable cytopathic effects in the infected BME/CTVM23, RSE/PILS35 and IDE8 cell lines were observed, starting from 8, 6 and 7 dpi, respectively. The diameters of the uninfected BME/CTVM23 and IDE8 cells in the Giemsa-stained cytocentrifuge smears ranged from 10 to 20 µm (Figure 1A,B), while the uninfected RSE/PILS35 cells ranged from 10 to 50 µm (Figure 1C). Pleiomorphic rickettsiae-like bacteria were observed in the cytoplasm of the tick cells, after infection with *R. raoultii* (Figure 1D–F). There was no observable difference in the size of the cells after infection with *R. raoultii*. Rickettsiae-like bacteria were observed in all the infected cultures, starting from 3 dpi. However, at this point, not many cells were infected in the BME/CTVM23 cultures and most of the bacteria were still extracellular. All the cells in both the BME/CTVM23 cultures appeared to be infected at 15 dpi. Large numbers of bacteria were seen outside the cells, together with many lysed cells in the cytocentrifuge smears by 18 dpi. For the RSE/PILS35 and IDE8 cultures, the rickettsiae-like bacteria were readily observed inside the cell cytoplasm at 3 dpi. More cells with bacteria inside the cell cytoplasm were seen at 7 dpi. Many infected cells were lysed and large numbers of bacteria were seen outside the cells after 10 dpi. By 15, 13, and 14 dpi, the culture medium in infected BME/CTVM23, RSE/PILS35 and IDE8 cultures, respectively, started to turn pink, suggesting that cells had begun to die.

### 3.3. Bacterial Infection Rates in Tick Cell Cultures

**Figure 2:**
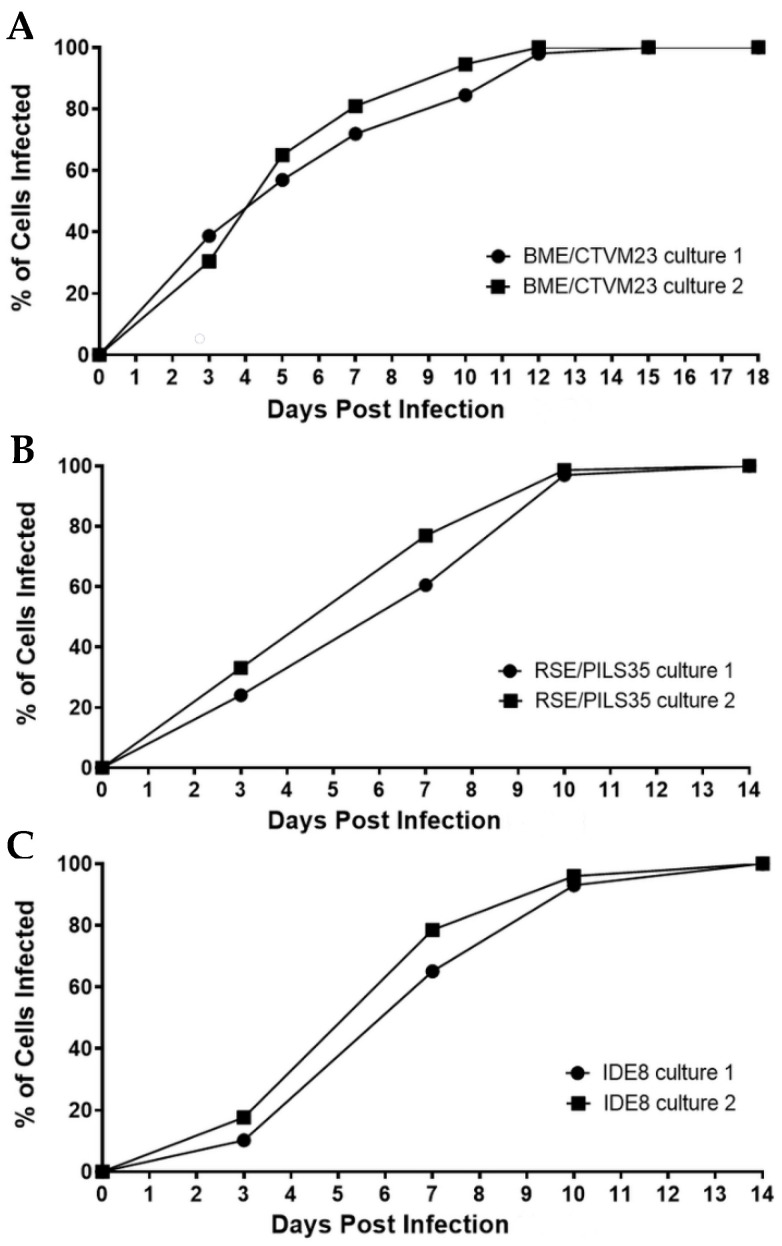
*Rickettsia raoultii* infection rate curves in two replicate cultures in each of the tick cell lines BME/CTVM23, RSE/PILS35 and IDE8. (**A**): *R. raoultii*-infected BME/CTVM23 cultures, (**B**): *R. raoultii*-infected RSE/PILS35 cultures, (**C**): *R. raoultii*-infected IDE8 cultures. The infection rates were calculated based on the percentage of infected cells observed among 200–300 cells counted in Giemsa-stained cytocentrifuge smears prepared at the indicated days post infection.

The percentages of infected cells in the two BME/CTVM23 cell cultures increased steadily from 0 to 12 dpi (Figure 2A). At days 5 and 10 dpi, the mean infection rates were 61.0% and 90.0%, respectively, and by 12 dpi, 100% of the cells from both the cultures were infected. Similarly, the numbers of infected cells in both the RSE/PILS35 cell cultures increased steadily from 0 to 10 dpi (Figure 2B), with mean infection rates at 3, 10 and 14 dpi of 28.5%, 97.0% and 100%, respectively. A lag phase was observed between 0 to 3 dpi in both the *R. raoultii*-infected IDE8 cultures (Figure 2C). This was followed by a steady increase in the percentage of infected cells to 10 dpi, when the mean infection rate reached 94.5%. Subsequently, 100% of the cells in both the cultures were observed to be infected at 14 dpi.

### 3.4. Replication Kinetics of R. raoultii in Tick Cell Cultures

**Figure 3:**
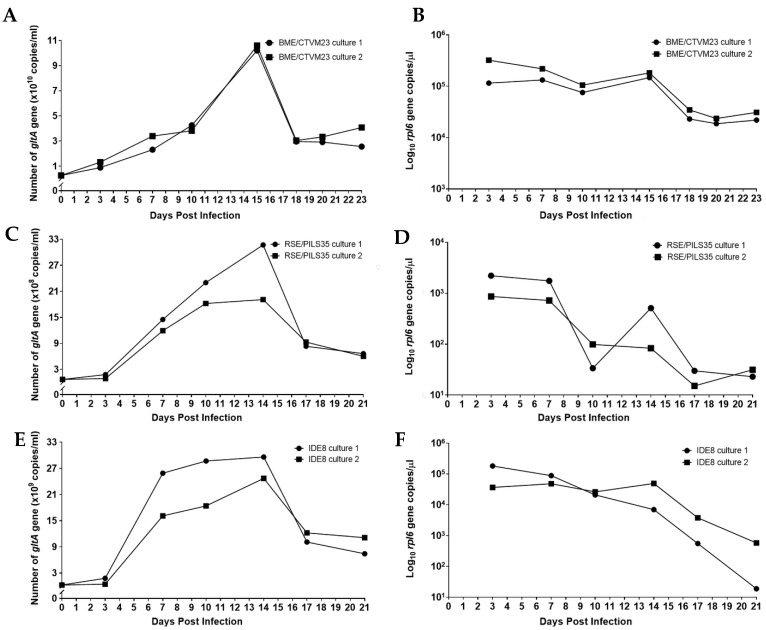
Replication kinetics of *Rickettsia raoultii* in tick cell lines. The growth curves for *R. raoultii* were established based on the copy number of rickettsiae-specific *gltA* gene determined for two replicate cultures each of the tick cell lines BME/CTVM23 (**A**), RSE/PILS35 (**C**) and IDE8 (**E**). Concurrently, the tick cell copy number was determined by amplification of the tick-specific rpl6 gene for each of the BME/CTVM23 (**B**), RSE/PILS35 (**D**) and IDE8 (**F**) cultures.

The numbers of *R. raoultii* were represented by the copy numbers of the rickettsiae-specific *gltA* gene target determined by qPCR. For the BME/CTVM23 cultures, approximately 2.63 × 10^9^ DNA copies of *R. raoultii* were used to initiate infections in both cultures, as indicated on 0 dpi. (Figure 3A). The curves for both the BME/CTVM23 cell cultures demonstrate an exponential increase from 0 to 15 dpi, and a subsequent declining phase from 15 to 18 dpi. The mean generation time for *R. raoultii* in the BME/CTVM23 cultures was 2.2 days. On 15 dpi, the highest mean copy number was recorded at 1.04 × 10^11^ copies/mL, representing an approximately 39.5-fold increase compared to the inoculum. Concurrently, a gradual decrease in the tick cell numbers, as represented by the *rpl6* target copy number, was observed in both the *R. raoultii*-infected BME/CTVM23 cell cultures from 3 to 15 dpi, followed by a steeper decline as the infected cells began to die (Figure 3B).

Approximately 6.85 × 10^7^ DNA copies/mL of *R. raoultii* were used to initiate infections in both the RSE/PILS35 cell cultures, as indicated at 0 dpi (Figure 3C). The curve for both the RSE/PILS35 cell cultures demonstrated an initial lag phase from 0 to 3 dpi. This was followed by an increase in the *gltA* target copies, from 3 dpi to 14 dpi, and a subsequent decrease, from 14 dpi to 17 dpi, in culture 1. On the other hand, in culture 2, the exponential increase in the *gltA* target copies, from 3 to 10 dpi, was followed by a stationary phase between 10 and 14 dpi, and a subsequent decrease from 14 dpi to 17 dpi. The *R. raoultii* generation times were calculated to be 2.6 and 1.6 days in cultures 1 and 2, respectively. On 14 dpi, the highest mean copy number was recorded at 2.54 × 10^9^ copies/mL, representing an approximately 37.1-fold increase compared to the inoculum. In parallel, the tick cell numbers remained fairly stable from 3 to 7 dpi in both the *R. raoultii*-infected RSE/PILS35 cell cultures, and were followed by a subsequent decline until 21 dpi (Figure 3D). However, in RSE/PILS35 culture 1, an increase in the copy number of the *rpl6* gene was observed at 14 dpi.

For the IDE8 cells, approximately 2.46 × 10^8^ DNA copies of *R. raoultii* were used to initiate infections in both the cultures, as indicated on 0 dpi (Figure 3E). The growth curves for both the IDE8 cell cultures demonstrate an initial lag phase from 0 to 3 dpi. This was followed by an exponential increase from 3 to 7 dpi, a subsequent stationary phase from 7 to 14 dpi, and a declining phase from 14 to 17 dpi, which remained low until 21 dpi in both the cultures. The mean generation time for *R. raoultii* in the IDE8 cultures was 0.9 day. On 14 dpi, the highest mean copy number was recorded at 2.71 × 10^10^ copies/mL, representing an approximately 110.1-fold increase compared to the inoculum. Simultaneously, a gradual decrease in the tick cell numbers, as represented by the *rpl6* target copy number, was observed in both the *R. raoultii*-infected IDE8 cell cultures, from 3 to 14 dpi, followed by a steeper decline until 21 dpi, as the infected cells began to die (Figure 3F).

## 4. Discussion

The data presented in the present study reveal that *R. raoultii* is able to infect and propagate in the *R. microplus*-derived BME/CTVM23, *R. sanguineus*-derived RSE/PILS35, and *I. scapularis-*derived IDE8 cell lines. The BME/CTVM23 cell line was selected for study because the *R. raoultii* strain used was isolated in this line [21], and the RSE/PILS35 cells were used because *R. raoultii* has previously been isolated in another *R. sanguineus* cell line [24]. *I. scapularis* cell lines are known to be permissive to infection with North American *Rickettsia* spp. [32,33,34,35,36]; the IDE8 line was used to determine whether *I. scapularis* cells were able to support replication of the Eurasian species *R. raoultii*. The presence of noticeable cytopathic effect was observed in all the infected tick cells in this study. However, a previous study [21] has shown that *R. raoultii* caused almost no cytopathic effect in primary *Dermacentor marginatus* cell cultures despite, or perhaps because of, this species being one of the main arthropod vectors of the bacterium. 

The infection rate curves showed that the percentage of infected cells in all three cell lines increased steadily from less than 40% at 3 dpi to 100% of the cells at the end of the observation period. Since the quantification of bacteria from Giemsa-stained cytocentrifuge smears is difficult, due to the variable number of bacteria infecting a single cell, it is necessary to perform qPCR alongside to determine the growth of bacteria in the infected cells.

Generally, the replication kinetics for bacteria can be represented by the following four common phases: the lag phase, exponential phase, stationary phase, and death phase [37]. These growth phases could be seen in both the infected IDE8 cultures and one of the infected RSE/PILS35 cultures. Similar replication kinetics of *R. raoultii* and *Rickettsia rickettsii* bacteria were observed in Vero cells, in which all four phases were observed [38,39]. In contrast, the stationary phase was not detected in either of the infected BME/CTVM23 cultures and one of the RSE/PILS35 cultures. For intracellular bacteria, such as *R. raoultii*, the death phase may occur when all the cells in the culture are infected and they begin to die. This could be observed in the decline of the tick cell copy numbers, and the onset of the bacterial death phase after 100% of the cells were infected in all three cell lines. The high bacterial numbers appear to have promoted rapid cell death and prevented the occurrence of a stationary phase in the BME/CTVM23 and RSE/PILS35 cultures. 

An initial lag phase in the *R. raoultii* copy numbers early in the infection was observed in both the infected RSE/PILS35 and IDE8 cultures. This observation may represent the adaptation of the bacteria to the host cell during the lag phase. The presence of a lag phase after the introduction of the bacterial inoculum was consistent with other studies of replication kinetics for different *Rickettsia* spp. in mammalian cell lines. These include lag phases of 7 days for *R. raoultii* [38], 2 days for *R. helvetica* [40], and 1 day for *R. rickettsii* [39] during infection of Vero cells, 6 days for *R. raoultii* during infection of L929 cells [38], and 7.5 h for *R. prowazekii* during infection of chicken embryonic cells [41]. The variable lengths of the lag phase for the different rickettsial species may be influenced by how the bacteria adapt to the different cell lines and the culture conditions. In addition, the length of the lag phase may also be dependent on the bacterial growth phase from which they were isolated for use in the infection [42]. On the other hand, the lag phase was not detected in either of the infected BME/CTVM23 cultures. The absence of a lag phase may be because the bacteria were originally propagated in BME/CTVM23 cells, hence they were already accustomed to growth in this cell line. 

The mean lengths of the exponential phase for *R. raoultii* infection in tick cells, ranging from four to twelve days, were observed to be longer than the reported lengths of the exponential phase during *R. raoultii* and *R. slovaca* infections in mammalian cells [38,43]. Apart from IDE8, the generation times for *R. raoultii* in the infected tick cultures ranged from 1.6 to 2.2 days, which are also greater than the generation times reported for *R. raoultii* and *R. slovaca* in mammalian cells, which ranged from 20 to 22 h [40,43]. The discrepancy observed in the growth rate of *R. raoultii* could be due to two possible reasons. Firstly, the infected tick cell cultures were maintained at a lower temperature compared to the usual incubation temperatures for the infected mammalian cells in previous studies, which could have influenced the growth rate of the bacteria. A recent report showed that *Candidatus* Rickettsia vini induced cell death in tick and Vero cells at approximately the same rate at incubation temperatures of, respectively, 28 °C and 32 °C [44]. Secondly, there may be possible variation in the interaction between the rickettsiae and the different cell lines. 

The increase in *R. raoultii* numbers observed during infection also varied between the tick cell lines tested in our study. The overall increase in bacterial numbers, compared to the starting inoculum in the IDE8 cultures, appeared to be greater than in the BME/CTVM23 and RSE/PILS35 cultures (i.e., 101.1 times vs. 39.5 and 37.1 times). This observation suggests that *R. raoultii* were able to multiply to higher numbers in the IDE8 cells. Furthermore, a stationary phase lasting up to seven days could be observed in the infected IDE8 cultures, and there was only a marginal decline in the tick gene copy numbers during this phase. This suggests that IDE8 cells may be more tolerant than the other two cell lines of heavy bacterial burden before the onset of cell death in the culture conditions used. 

In conclusion, we have demonstrated that *R. raoultii* can infect and propagate in BME/CTVM23, RSE/PILS35 and IDE8 cell lines, representing three species of metastriate and prostriate ticks not known to harbor this bacterium in nature. We have further demonstrated the growth kinetics of the bacteria in these cell lines. Our observations include a longer exponential phase and generation times, as well as higher bacterial multiplication levels in tick cell lines as compared to the observations in mammalian cells in previous studies. Further experiments, however, are necessary to examine the influence of incubation temperatures on bacterial growth. When the infected tick cell cultures were maintained under the same culture conditions, the IDE8 cells also appeared to be able to tolerate higher *R. raoultii* burdens and multiplication levels than the two metastriate tick cell lines. Further investigations will be necessary to determine if similar observations are made under different culture conditions, and to examine if the genotypic and phenotypic differences between the tick cell lines influence *R. raoultii* growth rates.

It is also important to note that the tick cell lines used in this study did not originate from the natural vector of *R. raoultii* and, therefore, do not represent the natural host cells of the bacteria. Nevertheless, these cell lines will still be useful for investigating arthropod–pathogen interactions, especially in the absence of continuous cell lines from any of the natural arthropod vectors.
